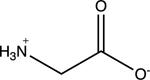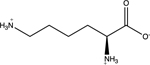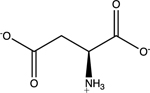# Light up My Life: An Active Learning Lab to Elucidate Conductive Properties of Electrolytes

**Published:** 2019

**Authors:** Christina Lee, Nay Chi P. Naing, Daisy Herrera, Gerardo Aguirre, Brandon Rodriguez, Jared Ashcroft

**Affiliations:** 1 Division of Natural Science, Pasadena City College, Pasadena, USA; 2 NASA Education, Jet Propulsion Laboratory, Pasadena, CA, USA

**Keywords:** Electrolytes, Acid-Base Theory, Biomolecules

## Abstract

Understanding molecular structure and its influence on chemical reactivity is a fundamental component in Chemistry curriculum. For example, acidic protons ionize, or ionic solids dissociate to form charge, inducing electrolyte properties depending on molecular structure. An active learning lab is designed to demonstrate connection between electrolyte behavior and structure of various molecules. Experiments are shared to show interdisciplinary aspect of electrolytes within biology and chemistry. Specifically, how biomolecules exhibit electrolyte behavior due to chemical composition.

## 1 Introduction

**Figure 1.**
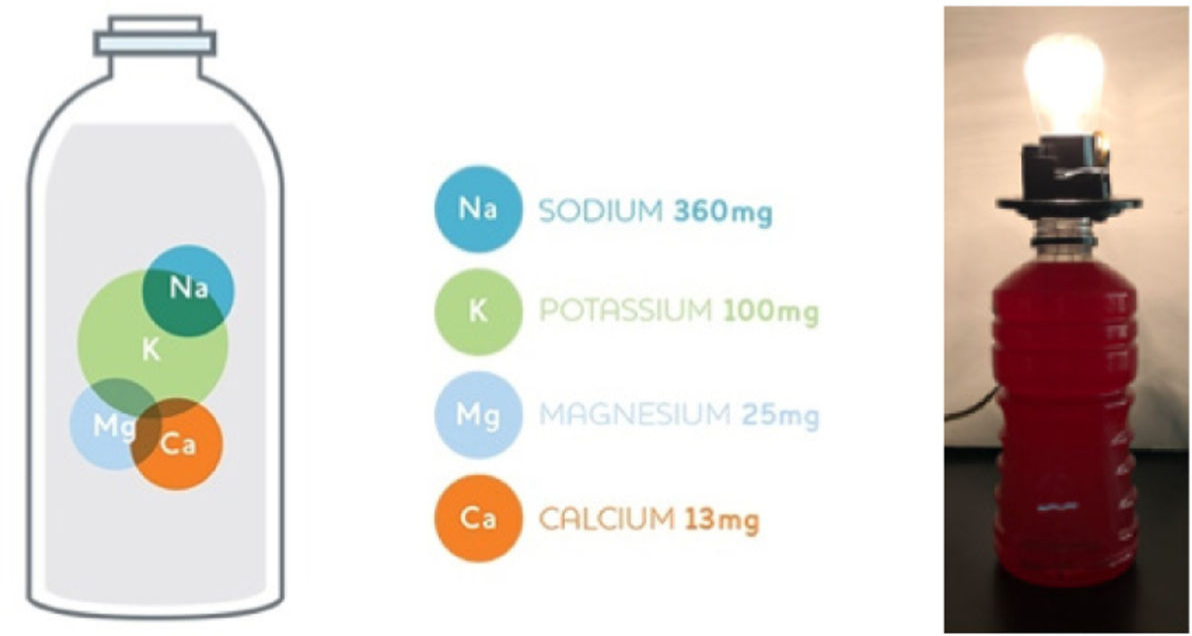
Electrolytes in sports drinks cause light bulb illumination

Sports drinks aim to replenish electrolytes in the body to increase athletic endurance. However, do athletes, especially young children, need drinks that contain large doses of sugar and subsequent calories? Can we get electrolytes from alternative, more nutritious sources? To get this answer, we must understand what an electrolyte is. Electrolytes are compounds that separate into ions, creating an electrically conductive solution. For example, when a light bulb is placed in a sports drink, the charges (from electrolyte ions) in the drink allow for an electric current to flow from an external energy source to make the light bulb glow (Figure 1).

Understanding electrolyte properties sets the foundation for future, more complex chemical topics such as acid-base theory and chemical equilibrium. It is also a building block for biologists understanding of amino acid or carbohydrate structure (Schank, 2019). Therefore, a lesson on electrolyte properties and the science behind advertising electrolyte drinks was developed for a general chemistry course. This understanding leads to more informed parents to choose alternative nourishment to their athletes besides the use of sports drinks.

Typically, electrolyte theory is taught via formulaic problem-solving strategies that are unengaging to students. While traditional methods of lecturing have been institutionalized, it does not optimize student learning, which is enhanced by student-centered teaching pedagogy (Landis, 1998). An electrolyte lab was developed to promote active learning in which students are encouraged to apply theoretical chemistry conceptual knowledge to experimental design (Ramdoniati, 2018). This type of learning promotes student engagement and leads to increase interest and success in general chemistry coursework (Spencer, 1999). Lab components focus on critical thinking and analysis as opposed to pre-contrived verification labs.

There are two main categories of electrolytes studied in this activity. The first is based on ionic solids that dissociate in water to form anions (negatively charged ions) and cations (positively charged ions). The most common example of this reaction is when table salt (NaCl) is placed in water, a dissociation occurs where sodium ion (Na^+^) and chloride (Cl^−^) are formed. These ionic based electrolytes are most commonly found in sports drinks.

The second category are electrolytes based on acid-base chemistry where proton (H^+^) transfer causes positively charged cations and negatively charged anions to form in water. This proton transfer leads to a solution that exhibits different magnitudes of electrical conductivity, thus yielding three types of electrolytes:

1. Strong electrolytes exhibit 100% ionization. For example, a strong acid, such as hydrochloric acid (HCl) completely ionizes in water to produce hydronium ion and chloride:
$${\text{HCl}}_{\text{(aq)}\;}+{\text{H}}_{2}{\text{O}}_{\text{(1)}\;}\to {\text{H}}_{3}{\text{O}}_{\text{(aq)}\;}^{+}+{\text{Cl}}_{\text{(aq)}\;}^{-}$$
2. Weak electrolytes partially ionize as demonstrated by acetic acid reaction with water to form acetate and hydronium ion. This is an equilibrium, whereas it can proceed either towards the products or the reactants, depending on reaction conditions:
$${\text{CH}}_{3}{\text{COOH}}_{\text{(aq)}\;}+{\text{H}}_{2}{\text{O}}_{\text{(1)}\;}⇌{\text{CH}}_{3}{\text{COO}}^{-}{}_{\text{(aq)}\;}+{\text{H}}_{3}{\text{O}}^{+}\text{(aq)}\;$$
3. Non-electrolytes, such as solid glucose do not ionize when combined with water. Rather, dissolution of solid occurs:
$${\text{C}}_{6}{\text{H}}_{12}{\text{O}}_{6(\text{s})}\to {\text{C}}_{6}{\text{H}}_{12}{\text{O}}_{6(\text{aq})}$$

Strong electrolytes are symbolized using a one-way arrow to show complete ionization, whereas weak electrolytes are symbolized using a double-headed arrow, signifying equilibrium or partial dissociation taking place.

Electrolyte theory is the impetus towards understanding acid-base chemistry, amino acid structure and a myriad of other scientific concepts. This activity enables students to visualize electrolytes in food and categorize acids or bases into strong, weak or non-electrolytes. Once visual confirmation is performed, conductivity values will be used to distinguish electrolyte strengths in foods and biological molecules. Lastly, students will utilize experimental data to infer the chemical structure of biomolecules when combined with water and use this information to formulate an understanding of how organic functional groups exhibit unique electrolyte behaviour.

## 2 Experimental Design

### 2.1 Standard Electrolytes

**Figure 2.**
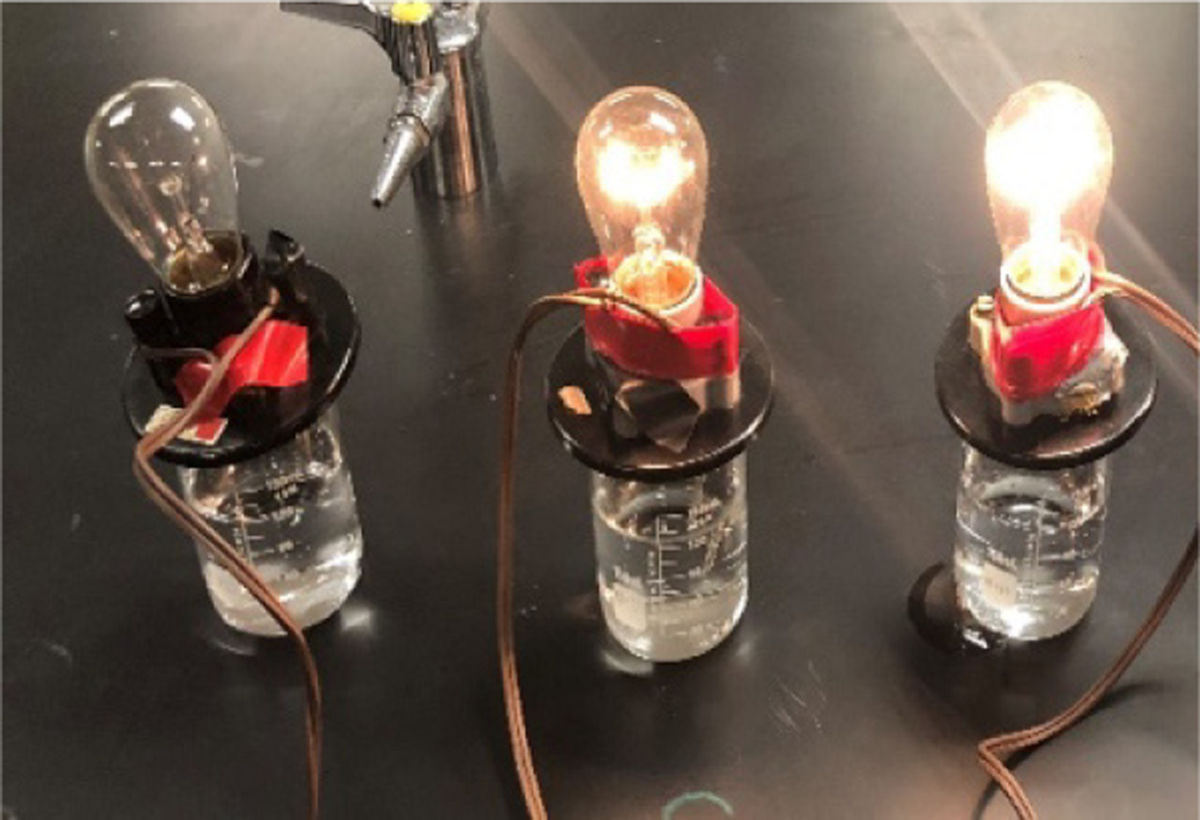
Extent illumination of a light bulb supported by water and solutions of benzoic (middle) and hydrochloric (right) acid

Equimolar solutions (0.125 M) of hydrochloric (HCl) and benzoic acids were prepared. A conductivity apparatus was used to visualize the illumination of light based on electrolyte formation (Figure 2). Distilled water was used as the standard non-electrolyte. A conductivity meter measured conductance in millisiemens (mS) to quantify conductivity differences between the strong, weak and non-electrolytes (Table 1). It was observed that HCl was the strongest electrolyte due to greatest illumination and highest conductivity (12.44 mS). Benzoic acid had a faint glow and relatively low (0.95 mS) conductivity, which demonstrates weak electrolyte behavior. Comparing illumination and conductivity values of hydrochloric and benzoic acid gives an indication of what strong versus weak electrolyte correspond in illumination and conductivity values.

### 2.2 Food Electrolytes

**Figure 3.**
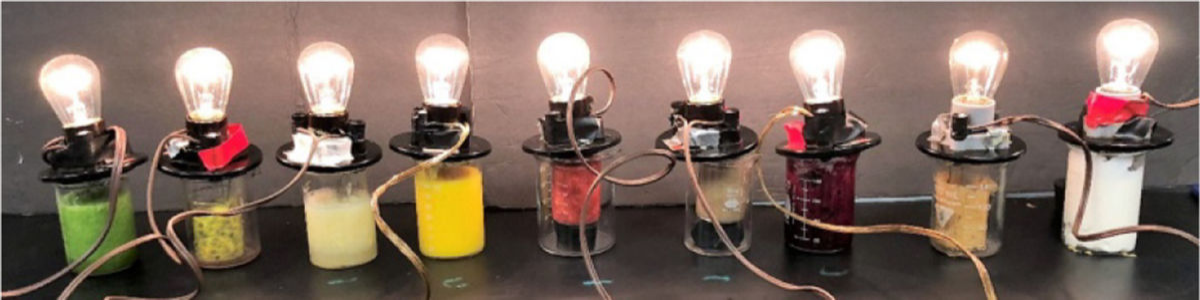
Light illumination from various food (left to right) cucumber, passion fruit, lemon, peach, strawberry, banana, beets, peanut butter, cream cheese

Students brought random food products to lab to investigate their electrolyte properties. Conductivity apparatus was employed to observe extent of electrolyte ability by emittance of light (Figure 3). Initial foods revealed strong electrolyte behavior. To connect food electrolytes to sports drinks, ingredients found in each were researched. The minerals in both food and sports drinks were found to be elemental ions causing light bulb illumination, showing sports drinks and healthier foods contain similar electrolyte producing substances. Students were tasked with finding disparate food items that exhibited strong, weak and non-electrolyte properties.

**Figure 4.**
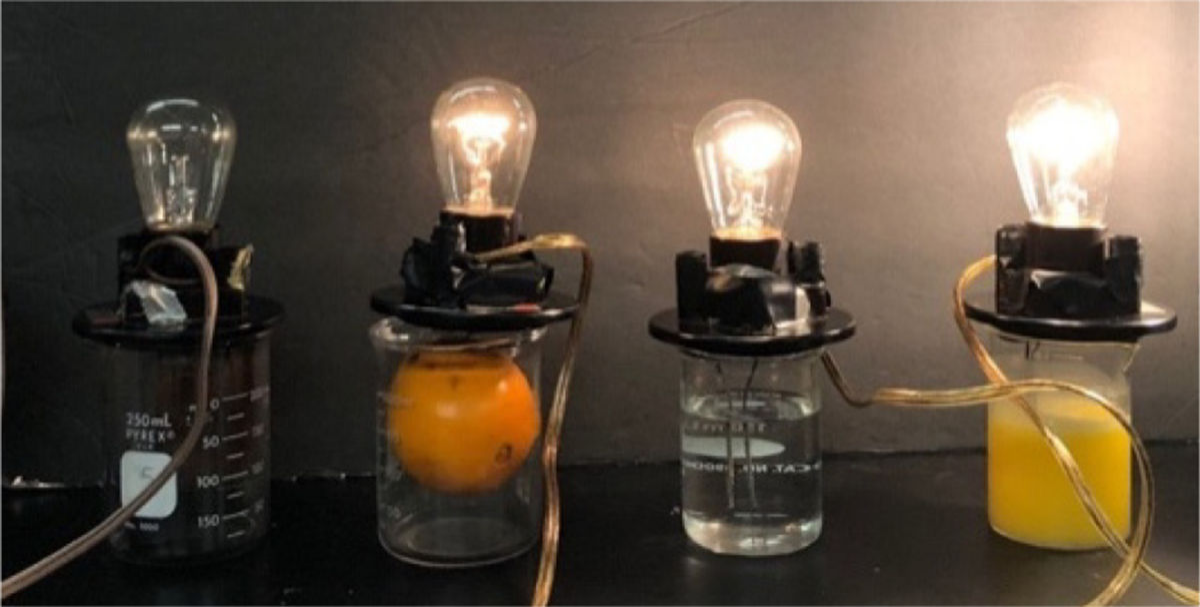
Light illumination from (left to right) chocolate, orange, vinegar, orange juice

Students chose the following food as non, weak and strong electrolytes for further study; chocolate, an orange, vinegar and orange juice. As shown in Figure 4 and Table 2 chocolate displayed non-electrolyte, vinegar weak electrolyte and the orange juice strong electrolyte behavior. To gain an understanding of how chemical structure affects electrolyte behavior, students researched ingredients of each food and postulated one compound that could be responsible for illumination: 1) theobromine in chocolate,2) acetic acid in vinegar and 3) citric acid in oranges. Interesting, when performing the illumination experiment on an orange (conductivity could not be measured) verse orange juice, the orange glow was less then orange juice and fainter then vinegar. This demonstrates effect that concentration has on electrolyte behavior. The aqueous environment in the native orange does not provide a sufficiently concentrated solution of citric acid for conductance. Therefore, the orange juice exhibits stronger electrolyte behavior.

To increase understanding of electrolyte properties in relation to organic structure chocolate, vinegar and orange juice can be compared with standard compounds in **Part 1**. Students should notice the common -COOH carboxylic acid functional group and determine that the proton attached to oxygen is acidic, can ionize and therefore lead to electrolyte behaviour. Analysing structure of chocolate, which does not contain -COOH functional group explains its non-electrolyte manner. The 3:1 -COOH ratio in citric verse acetic acid molecules explains the stronger electrolyte behaviour of orange juice verse vinegar. This is an oversimplification of electrolytes, but for the purpose of this introductory experiment, will help students gain a rudimentary understanding of relationship between structure and electrolyte behaviour.

The food study requires students to combine scientific literacy with experimental data to determine various electrolytes in food and demonstrate knowledge of electrolytes by correctly identifying substances that cause electrolyte behaviour. By comparing the electrolyte behaviour of a non, weak and strong electrolyte and drawing the structures of each, an understanding of organic structures/functional groups will lead them to the next component of the experiment.

### 2.3 Assessments Part 1 and 2

Students were asked to visualize, quantify and demonstrate their understanding of electrolytes by identifying and writing correct non, weak and strong electrolyte reactions. An assessment was done on students work based on the following rubric (Table 3):

Assessments were performed in the following order: 1) A pre-quiz was given one day prior to lab after a short lecture on electrolytes was given in class. Students were asked to identify and correctly write a non, weak and strong electrolyte reaction. 2) Students performed the electrolyte lab and immediately were given the same quiz as the day before to see if improvement occurred. 3) Two days after the lab, students were asked to identify and write non, weak and strong electrolytes that were part of the experiment as well as different electrolytes on an exam. The quizzes and lab sheets have been attached as supplemental materials.

**Figure 5.**
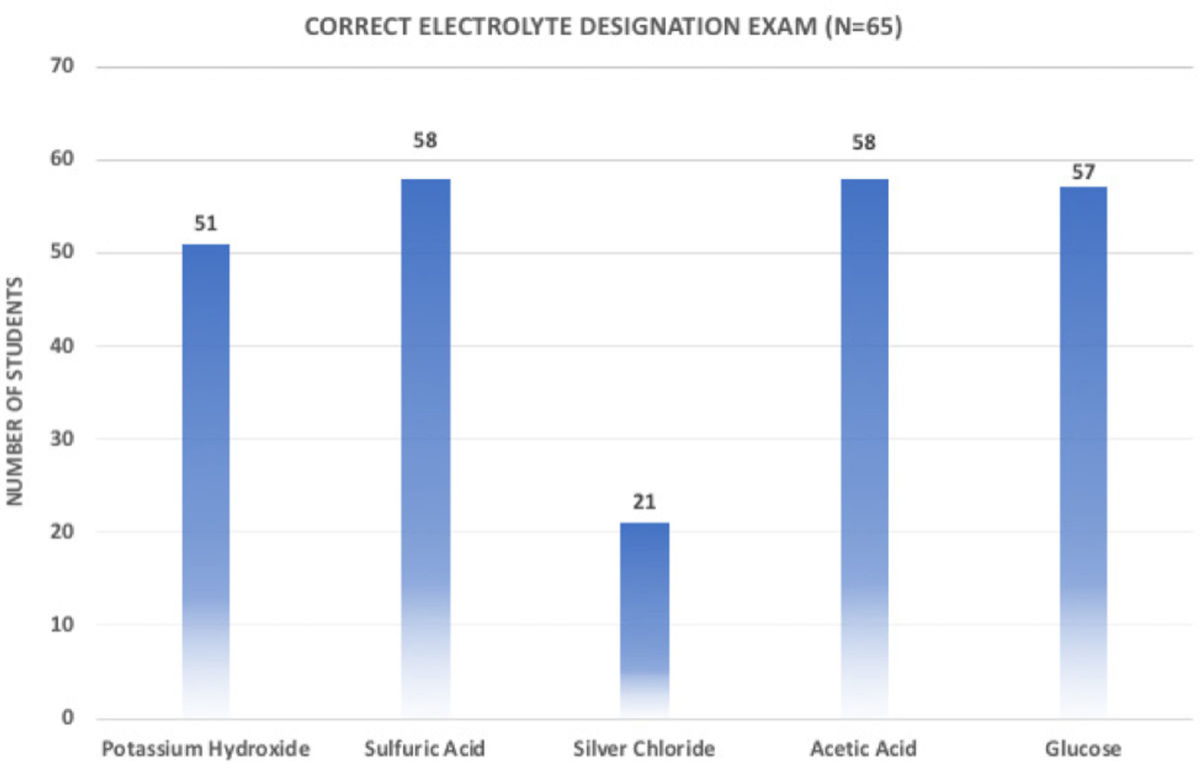
Identifying electrolyte exam

Students performed well on identifying types of electrolytes on the exam (Figure 5). Potassium hydroxide and sulfuric acid were not previously discussed, yet students demonstrated ability to identify strong acids (89%) and bases (78%) as strong electrolytes. Silver chloride was added to the exam questions to see if students could identify a non-electrolyte salt. As shown below, students were not able to relate the non-electrolyte salt to electrolyte behaviour. Glucose and acetic acid, two electrolyte standards in the experiment were correctly identified by approximately 90% of the students.

**Figure 6.**
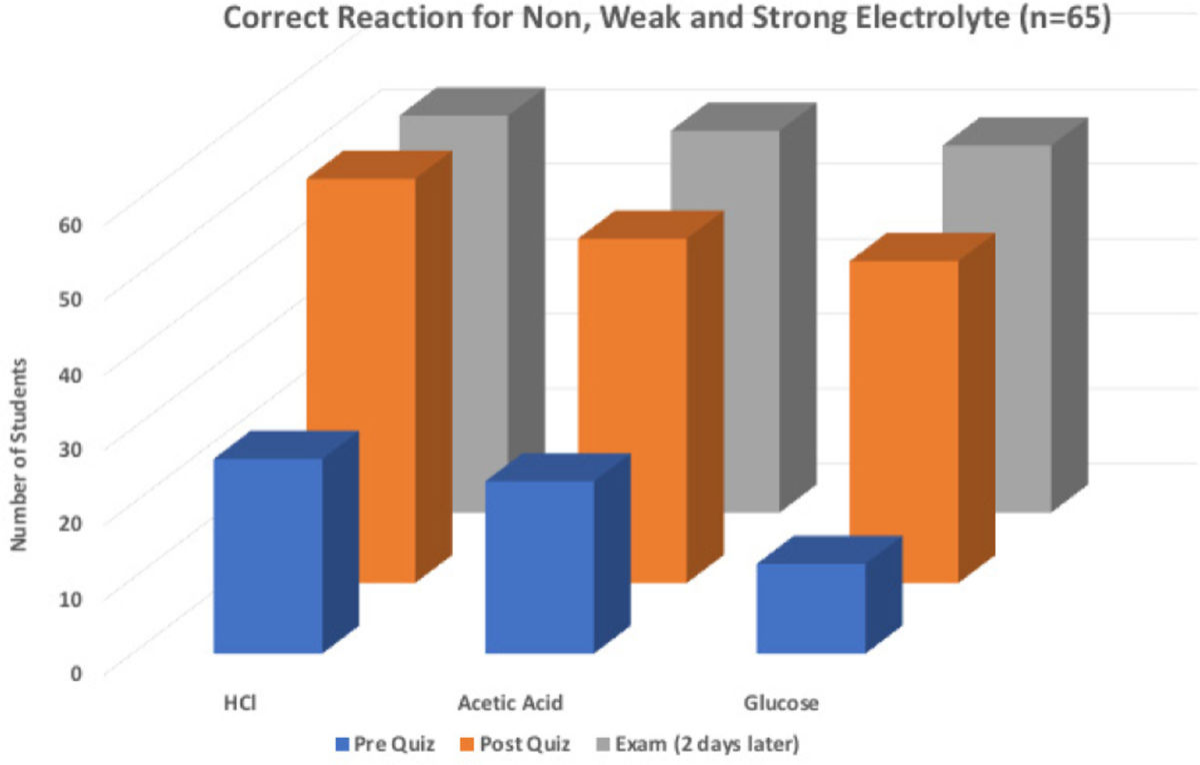
Non, weak and strong electrolyte reaction

The second part of the assessment was the ability to correctly identify and draw the non, weak and strong electrolyte reactions. As shown in Figure 6, most students (less than 50%) were unable to write the correct reactions prior to the activity. Immediately following the lab, students were given the same question as a post quiz, showing improvement writing the non, weak and strong electrolyte reactions (greater than 80%). Results from exam two days later showed student retention of the knowledge gained from electrolyte experiment. Lastly, overall grades based on rubric in Table 3 showed student increased from pre-quiz (1.86), to post quiz (2.98), to the exam (3.13). For comparison, previous semesters when electrolyte theory was done via traditional lecturing, overall class average based on rubric was 2.62. This data suggests students were able to gain an understanding of electrolyte reactions using this experiment.

### 2.4 Biomolecule Electrolytes

Interdisciplinary nature of science enhances curiosity and engagement of students (Mammadov, 2016). Electrolyte lab presented is ideal in demonstrating interconnectivity between chemistry and biology. Biomolecules have structure that affect chemical properties, such as pH, which in turn related to chemical concepts like acid-base theory and electrolyte behavior. Students wrote a list of biomolecules; amino acids and carbohydrates were popular selections. Research was conducted on structure of these biomolecules, and on vitamin C. A hypothesis was formulated to predict the category of electrolyte for each biomolecule (Table 5) based on data and structure of compounds from previous lab activities. Electrolyte hypothesis in Table 5 was based on student suggestion.

**Figure 7.**
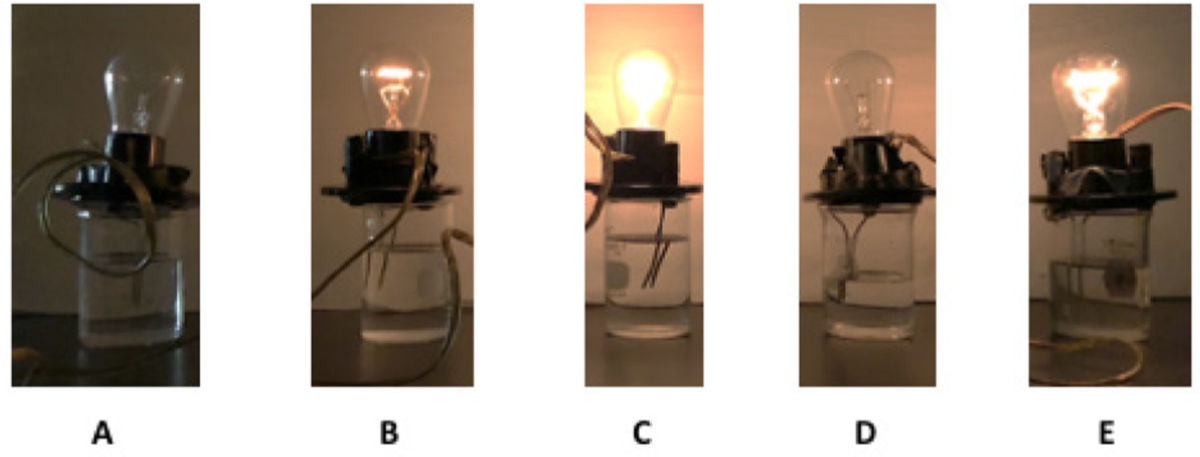
Electrolyte visualization of biomolecules A) glycine B) aspartic acid C) lysine D) glucose E) ascorbic acid

By identifying name, structure and possible electrolyte properties, students are presented with an engaging introduction to biomolecules. Once they have completed the research and identification process, a set of biomolecules is given to analyze electrolyte properties, congruent to parts 1 and 2 of the experiment. Once again, using the conductivity apparatus and meter, students visualize light output (Figure 7) and conductivity values (Table 4) of the biomolecules.

Once electrolyte strength has been determined, students were given an assignment to circle acidic protons responsible for electrolyte behavior and to draw correct structures of biomolecules added to water. For carbohydrates, since they are mostly non-electrolytes, it was determined that in aqueous media, they retain the same molecular structure as when in solid phase, merely dissolving rather than ionizing. However, amino acids bring several challenging concepts to the forefront that can be introduced as a precursor for future chemistry/biology courses.

Amino acids are essential building blocks of protein that combine to form a polypeptide chain. Each amino acid contains a carboxyl group (-COOH), an amino group (-NH_2_) and a unique R group which distinguishes it from other amino acids. At physiological pH (7.2 to 7.4), the amino group exists as a positively charged cation and the carboxyl group a negatively charged anion. The three amino acids studied in the second portion of this lab are glycine (nonpolar), lysine (+ R group) and aspartic acid (- R group). According to Thomas E. Needham in his paper *The Solubility of Amino Acids in Various Solvent Systems*, “the solubility, behavior of those amino acids studied was a function of the constant effect of the amino/carboxylic acid portion of the molecule and the independent interactions of the remaining neutral portion of the molecule” (Needham, 1970). This is most obvious for the nonpolar amino acid Glycine as its R groups has least interaction with water, relying mostly on amino/carboxyl groups. For charged R group amino acids, electrolyte properties are difficult to conceptualize. Therefore, the assignment was for students to observe electrolyte behavior of standard solutions in order to compare and determine electrolyte behavior of amino acids. Ultimately, students were expected to use electrolyte behavior to establish structure of amino acids when added to water.

Data showed glycine as a non-electrolyte, aspartic acid as a weak electrolyte and lysine as a strong electrolyte (Table 4). In comparing structure of glycine to standard acetic acid, the carboxylic acid functional group is on both. Students should ascertain that since the -COOH group ionized to form -COO^−^ and H^+^ in acetic acid, it should also ionize for glycine biomolecule. In comparing glycine to aspartic acid, observing illumination and conductivity occurred should lead to the conclusion that carboxylic acid functional groups do lose protons to form ionized products that lead to electrolyte behavior. At this point, students should begin questioning the difference in amino acid electrolyte behavior.

To better understand carboxylic acid and amine behavior, students analyze three amino acids, glycine, lysine and aspartic acid. Glycine shows non-electrolyte behavior which could be concluded that no proton ionizes from glycine. Both aspartic acid and lysine exhibit electrolyte properties due to illumination and conductivity. First, comparison of aspartic acid and glycine lead to an analysis that since both have a carboxylic acid functional group, both should exhibit electrolyte behavior. Further analysis shows lysine with electrolyte properties, which has an amine R group. Students conclude that amine functional groups do effect electrolyte properties as well. Since hydrogen on a neutral nitrogen cannot be donated in water, the electrons on nitrogen must accept an electron and act as a base.

To understand this behavior, it is important to give a discussion on isoelectric point (pI). The isoelectric point is the pH at which a molecule, in this case amino acids, carry no net electrical charge. Lack of conductance in glycine demonstrates that formation of charge alone is not sufficient for conductance. Since glycine at the isoelectric point (pI = 5.97) has a distinct positive (-NH_3_^+^) and negative (COO^−^) present within the molecule, it will remain neutral overall, unable to support electrical conductance. At this point, the amino acid acts as a Zwitterion, a molecule or ion that has separately positively or negatively charged groups. The additional carboxylic acid R group on aspartic acid and amine R group on lysine allow the molecules to undergo additional ionization in water. For aspartic acid, the second -COOH group protonates water molecules to form negatively charged structure shown in Table 6, while also producing separate hydronium ions (H_3_O^+^). Thus, distinct positively and negatively charged ions form. These ions, like Na^+^ and Cl^−^ are required to support conductance. Similarly, the second amine group in lysine acts as a base and ionizes water to for the positively charged structure shown in Table 6, while forming separate hydroxide (HO^−^) ions. As an extension to the experiment, students could be asked to research the isoelectric points of aspartic acid (pI = 2.77) and lysine (pI = 9.74) and design an experiment to make the solutions of these amino electrically neutral. Students would need to realize that when pH = pI, the solution will become electrically neutral, and subsequent addition of strong acid to aspartic acid or strong base to lysine while monitoring with the conductivity apparatus will lead to success in this experiment.

**Figure 8.**
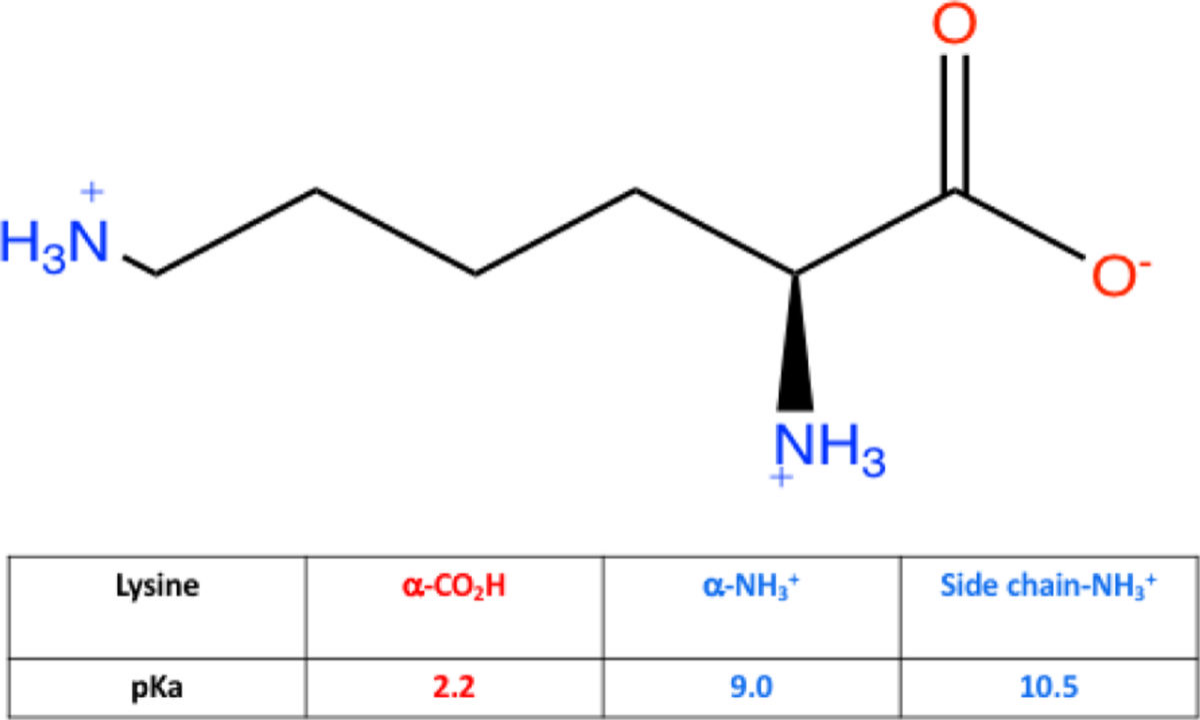
Structure and pKa values of lysine in water (pH~7.5)

To take this concept further, extent of protonation can be assessed using pKa values, which are used to measure acid strength in a compound. The lower the pKa, the stronger the acid. Depending on functional group, acid strength can be high, such as for carboxylic acids (pKa~4) or low for functional groups like neutral amines (pKa>35). Comparing the pKa values to pH shows whether a proton is attached to the molecule (pH<pKa) or not attached to the molecule (pH>pKa). Knowing the pH of solution and pKa of acidic sites on a molecule allows for determination of most probable molecular structure and comparison of structure to electrolyte strength. pKa also determine the isoelectric points for amino acids. For example, the pKa of carboxylic acid and amine on glycine is 2.34 and 9.60. The isoelectric point will be halfway between or the average of the two pKa values (pI = ½(2.34 + 9.60) or 5.97). Here is research and data students collected for lysine:

Students obtained the structure and pKa values for lysine acidic sites. After data collection above, students are asked to draw the form of lysine, as well as write an explanation for its electrolyte properties. In order to do this properly, students measure the pH for lysine solution (pH~7.5). Using pH and pKa comparison, an explanation of which protons are attached to specific acidic sites on lysine and which are no longer bonded can explain the strong electrolyte behavior of lysine solution. Since the pH is above 2.2, the carboxylic acid end of lysine ionizes (forming COO^−^) but since the pH is below 9.0, both the amine end and R group side chain exist as the protonated amine (NH_3_^+^) as shown in Figure 8. Build-up of charge in solution leads to strong electrolyte properties. This is an oversimplification of what is occurring, but for a first semester general chemistry course, sufficient for simple understanding of acid-base behavior and electrolyte outcomes.

**Figure 9.**
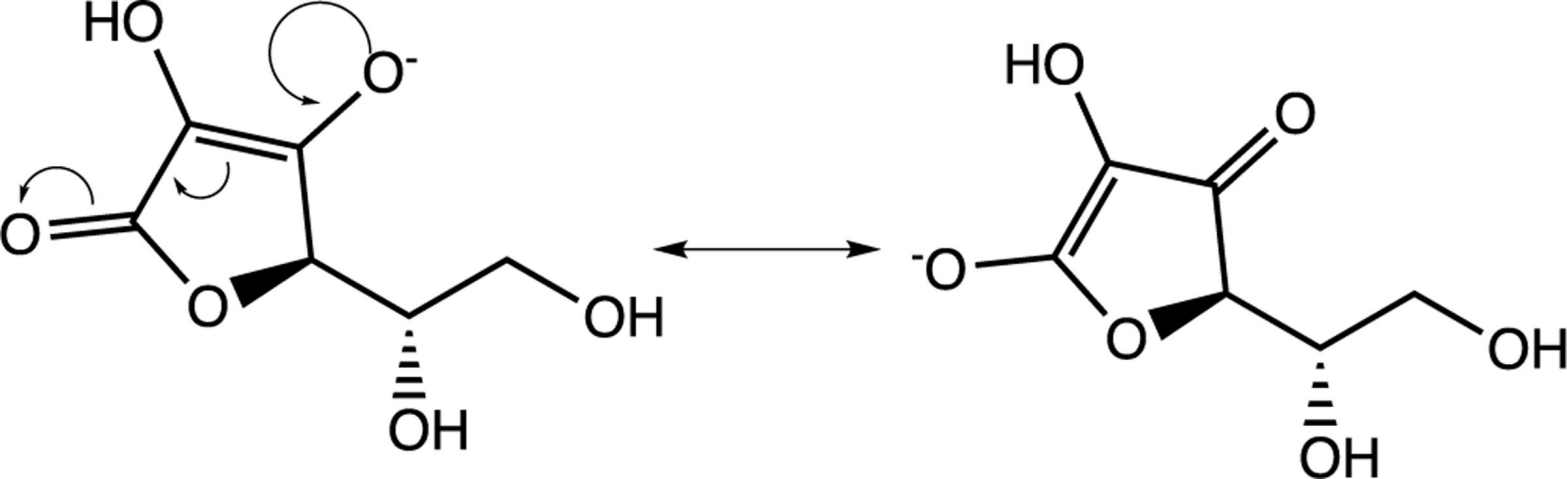
Resonance stabilization of ascorbic acid (vitamin C)

The last topic to be explored by students is data collected for ascorbic acid (vitamin C). The structures of both glucose and ascorbic acid only contain alcohol (-OH) functional groups (Table 4), yet ascorbic acid displays electrolyte behavior (Figure 7, Table 5). Students are asked to explain this discrepancy, since previously it was shown similar functional groups display same acid-base/electrolyte behavior. Analysis of ascorbic acid structure leads to ionization of ascorbic acid due to resonance stabilizationas shown in Figure 9. This resonance stabilization leads directly to electrolyte properties observed by vitamin C.

## 3 Student Feedback

Throughout the experiment, students were asked for feedback on increased understanding of electrolytes. The student rating after the lab was 3.4/5.0. Most of the feedback was frustration at not being told how to do the lab and that determining acidic protons on the biomolecules was challenging. Students were satisfied with the aspect where they had to write the non, strong and weak electrolyte reactions.

The following lecture, students were presented the theory behind electrolyte lab, using data from their experiment as examples. When asked if doing the lab beforehand helped in understanding, the scores on a five-point scale raised to 3.9/5.0. This demonstrates that performing active learning electrolyte lab before utilizing traditional lecture is optimal in engaging students and leads to increased performance analyzing molecular structure and its effect on electrolyte behavior.

## 4 Conclusions

A first semester general chemistry experiment was designed to investigate electrolyte properties. The active learning, connection to real life and interdisciplinary aspect of the experiment led to students being more engaged and interested in electrolyte chemistry. The introduction into electrolytes using this activity is a bridge to more advanced acid-base chemistry. Demonstrating the biological applications of electrolytes is beneficial in showing students the interrelationship biology and chemistry share. Overall, students were introduced to electrolytes in food and were able to see that several foods have similar electrolyte properties as sports drinks. Students were also introduced to how chemical makeup can lead to chemical properties, specifically how electrolyte properties are dependent on the functional groups of amino acids and carbohydrates. At the conclusion of the activity, students showed increased improvement in their understanding of the relationship between chemical structure and electrolyte behavior.

## Supplementary Material

Ashcroft_Supplemental_materials

## Figures and Tables

**Table 1.** Conductivity value of standard electrolytes

Substance (0.0125M)	Electrolyte	Conductivity (mS)
DI water (H_2_O)	non	0
Benzoic Acid (C_6_H_6_COOH)	weak	0.95
Hydrochloric Acid (HCl)	strong	12.44

**Table 2.** Structure, conductivity and electrolyte type various food

Food (Ingredient)	Structure Main Ingredient	Conductivity (mS)	Electrolyte
Chocolate (Theobromine)	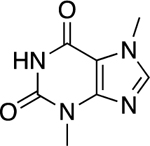	0	Non
Vinegar (Acetic Acid)	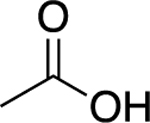	1.20	Weak
Orange Juice (Citric Acid)	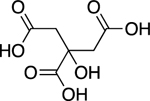	3.27	Strong

**Table 3.** Rubric to assess student understanding of electrolyte

	1-Unsatisfactory	2-Needs Improvement	3-Satisfactory	4-Master
Identifying Electrolyte	Student could not correctly identify types of electrolytes.	Student could correctly identify the standard electrolytes, but not identify electrolytes in food.	Student could correctly identify the standard electrolytes, and either a strong or weak electrolyte in food, but not both.	Student could correctly identify the standard electrolytes and weak/ strong electrolytes in food.
Electrolyte Reactions	Students could not correctly write electrolyte reactions.	Students could correctly write electrolyte reactants or products, but not both.	Students could correctly write reactant and products but used incorrect arrows and states of matter for electrolyte reaction.	Students correctly wrote electrolyte reactions.

**Table 4.** Name, structure, biomolecule and student electrolyte hypothesis

Structure and name	Biomolecule	Electrolyte Hypothesis
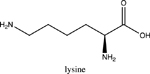	Amino Acid	Weak
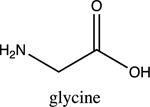	Amino Acid	Weak
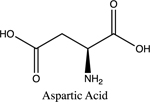	Amino Acid	Strong
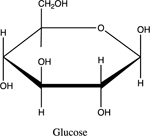	Carbohydrate	Weak
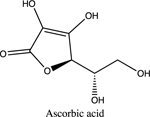	Vitamin	Weak

**Table 5.** Conductivity values of biomolecules

Biomolecule (3g / 50mL)	Conductivity (mS)	Electrolyte Strength
Glycine	0	Non
Aspartic Acid	0.65	Weak
Lysine	3.66	Strong
Glucose	0	Non
Ascorbic Acid	1.99	Weak

**Table 6.** Structure of amino acids in water

Glycine	Lysine	Aspartic Acid