# Supplementary material for: Light up My Life: An Active Learning Lab to Elucidate Conductive Properties of Electrolytes
Source: J Lab Chem Educ. Author manuscript; Available in PMC 2022 Aug 22. (PMC9395145)
Supplement: Ashcroft_Supplemental_materials [file NIHMS1056510-supplement-Ashcroft_Supplemental_materials.pdf]

Light Up My Life: Properties of Electrolytes

Pre-Lab Questions:

1. Write the reaction for the following:

   A. Hydrochloric Acid in Water:

   B. Acetic Acid in Water:

   C. Glucose ($C_6H_{12}O_6$) in Water:

2. Circle the molecule above that is a weak electrolyte.

3. Circle all the acidic hydrogens in glucose and glutamic acid below:

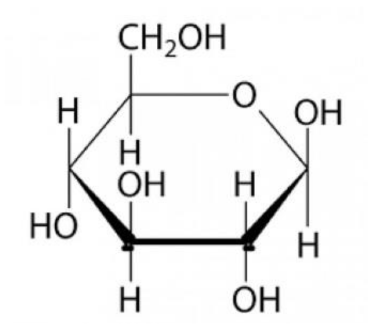

Glucose

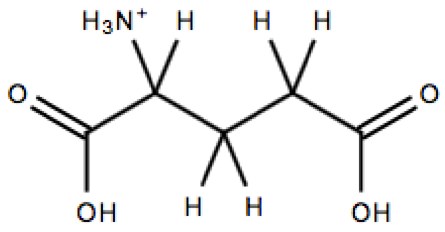

Glutamic Acid

Lab Questions:

Part 1: Standard Electrolytes

1. What does the glow of the light tell you about electrolyte type?

2. The standard electrolytes are water, acetic acid and hydrochloric acid. Put them in order of weakest to strongest electrolyte.

   _____________ < _____________ < ______________

3. Which conductivity would you expect to be highest for water, acetic acid and hydrochloric acid? Explain.

Part 2: Food Electrolytes

1. Label whether the following foods are strong, weak or non-electrolytes:

   A. Orange _____________________

   B. Vinegar _____________________

   C. Orange Juice _______________________

   D. Your Food _______________________ Electrolyte Type _________________________

   E. Your Food _______________________ Electrolyte Type _________________________

2. Choose two of the food items that have different electrolyte properties and explain why each of the foods displays those properties.

   A.

   B.

Part 3: Biomolecule Electrolytes

1. Choose three biomolecules. Write the name and draw the structure of each biomolecule in the boxes provided below. Based on parts 1 and 2 of the lab make a hypothesis on whether each biomolecule will be a weak, strong or non-electrolyte.

Name ____________________ Name ____________________ Name ____________________

Electrolyte ________________ Electrolyte ________________ Electrolyte ________________

2. Use the conductivity apparatus. Based on light illuminations, draw the structure of biomolecule that exists after placing in water. What type of electrolyte was each biomolecule based on your experiment.

Name ____________________ Name ____________________ Name ____________________

Electrolyte ________________ Electrolyte ________________ Electrolyte ________________

3. Choose one of the biomolecules and give a brief explanation of what happens when the biomolecule is placed in water.

Post-Lab Questions:

1. Write the reaction for the following:

   D. Hydrochloric Acid in Water:

   E. Acetic Acid in Water:

   F. Glucose ($C_6H_{12}O_6$) in Water:

2. Circle the molecule above that is a weak electrolyte.

3. Circle all the acidic hydrogens in glucose and glutamic acid below:

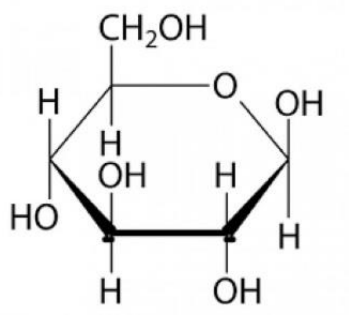

Glucose

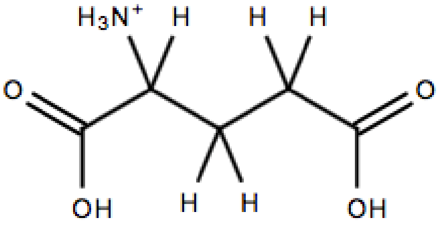

Glutamic Acid

4. On a scale of 1-5, 1 being not helpful, 5 being very helpful, How would you rate the effectiveness of this lab. Explain.

   Rating ______

   Explain:
